# The Hsp90 inhibitor NVP-AUY922-AG inhibits NF-κB signaling, overcomes microenvironmental cytoprotection and is highly synergistic with fludarabine in primary CLL cells

**DOI:** 10.18632/oncotarget.491

**Published:** 2012-05-18

**Authors:** Elisabeth Walsby, Lawrence Pearce, Alan K. Burnett, Chris Fegan, Chris Pepper

**Affiliations:** ^1^ Department of Haematology, Institute of Cancer and Genetics, Cardiff University, School of Medicine, Heath Park, Cardiff

**Keywords:** Hsp90, CLL, apoptosis, synergy, NF-κB

## Abstract

Heat shock protein 90 (Hsp90) is a molecular chaperone required for the stability and function of multiple over-expressed signaling proteins that promote growth and survival in cancer cells. Chronic lymphocytic leukaemia (CLL) is characterized by increased expression of several Hsp90 client proteins making it a potentially susceptible to Hsp90 inhibition. In this study we showed that the novel Hsp90 inhibitor NVP-AUY922-AG was cytotoxic to primary CLL cells in vitro (LD_50_=0.18μM±0.20). Importantly, its toxicity was preserved under cytoprotective co-culture conditions that rendered fludarabine ineffective. At the molecular level, NVP-AUY922-AG depleted the expression of multiple Hsp90 client proteins including Akt and activators of NF-κB, IKKα and IKKβ. Consistent with this inhibition profile, NVP-AUY922-AG resulted in decreased transcription of the NF-B target genes MCL1, CFLAR, BIRC5. In contrast, fludarabine significantly induced the transcription of MCL1 and BIRC5. Given the anti-apoptotic nature of these genes and the role they play in fludarabine resistance, we considered that the combination of NVP-AUY922-AG with fludarabine might resensitize CLL cells to the effects of fludarabine. In keeping with this hypothesis, the combination of NVP-AUY922-AG and fludarabine was highly synergistic (mean CI=0.110.06) and this synergy was enhanced in co-culture (mean CI=0.06±0.08). Furthermore, the combination maintained the decrease in MCL1, CFLAR and BIRC5 transcription suggesting that the ability of NVP-AUY922-AG to modulate expression of these genes may contribute to the efficacy of this drug under cytoprotective co-culture conditions and for its remarkable synergy with fludarabine. Taken together these findings indicate that Hsp90 inhibition is an attractive therapeutic strategy in CLL.

## INTRODUCTION

B cell chronic lymphocytic leukemia (CLL) is the most common leukemia in the western world and is characterized by an accumulation of monoclonal mature B cells within lymphoid organs, bone marrow and peripheral blood [1]. Microenvironments within the bone marrow and lymph nodes play a prominent role in CLL [1] as proliferation of CLL cells occurs in these centers [2]. It is now clear that CLL cell survival and activation is promoted by bone marrow stromal cells, follicular dendritic cells and T cells in vivo [1]. Furthermore, co-culture with bone marrow stromal cells in vitro can inhibit CLL cell apoptotic responses to purine analog chemotherapy through anti-apoptotic signals derived from CLL cell-stromal cell contact [3]. This microenvironment-derived cytoprotection against chemotherapeutic drugs likely contributes to treatment failure and relapse in CLL. Recently published gene expression profiling supports this view as lymph node-derived CLL cells showed a profile consistent with enhanced tumor proliferation and activation of the NF-κB pathway [2]. Other genes, including the inhibitor of apoptosis Survivin, are induced by NF-κB signaling via CD40-CD40L interaction [1] and in vivo Survivin-expressing cells are confined to the lymph node and pseudo follicles in the bone marrow [1].

Heat shock protein 90 (Hsp90) is part of the cellular chaperoning machinery that plays a role in maintaining protein functions including trafficking, post-translational stability and turnover of its protein substrates (clients) [4, 5]. Inhibition of Hsp90 function leads to proteasomal degradation of Hsp90 client proteins [4] and in theory results in simultaneous blockade of multiple oncogenic signaling cascades [5]. Cancer cells often contain elevated levels of Hsp90 [5] but the data on CLL cells is equivocal since there are conflicting reports on the relative level of Hsp90 expression in CLL cells and normal peripheral blood mononuclear cells [6, 7]. However, Hsp90 inhibitors have previously been shown to have activity in CLL cells [6] and the resulting toxicity was shown to partly attributable to the inhibition of NF-κB [8]. NF-κB is commonly up-regulated in CLL [9] and is maintained, at least in part, by interactions with the microenvironment [10]. Furthermore, NF-κB has been shown to be a therapeutic target in CLL [9, 11] but no specific NF-κB inhibitors are currently available for the treatment of this disease.

In this study we assessed the effect of the Hsp90 inhibitor NVP-AUY922-AG, as a single agent and in combination with fludarabine, on CLL cells under different cell culture conditions. We examined the effects of Hsp90 inhibition on Hsp90 client proteins and downstream signaling pathways and evaluated the potential for synergy with fludarabine.

## RESULTS

### IL-4 and co-culture with NTL and CD40L cells reduce spontaneous apoptosis in vitro CLL cell culture

**Figure 1:**
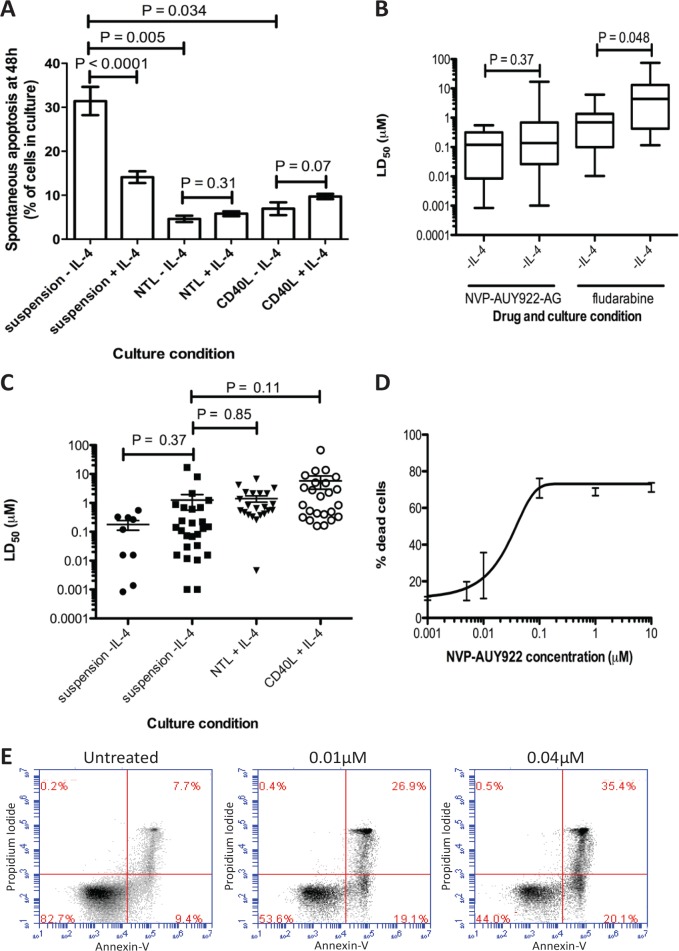
NVP-AUY922-AG is more cytotoxic that fludarabine, retains its activity in cytotoxic culture conditions and induces apoptosis in CLL cells in vitro A) Culture of CLL cells in vitro in the presence of IL-4 reduces the level of spontaneous apoptosis seen after 48h (P < 0.0001). NTL or CD40L expressing co-culture resulted in cytoprotection of CLL cells in culture (NTL P < 0.0001, CD40L P = 0.0003). B) LD_50_ values for CLL cells treated with NVP-AUY922-AG and fludarabine in vitro for 48h showed that cytotoxic effect of fludarabine was significantly reduced by the presence of IL-4 in culture (P = 0.048) while NVP-AUY922-AG was not. C) NVP-AUY922-AG retains cytotoxic activity on CLL cells in vitro in 48h treatments in suspension culture and in cyto-protective co-culture conditions. No differences in mean LD_50_ in CLL cells were significant. D) Dose response of CLL cells to NVP-AUY922-AG. Apoptosis was confirmed by annexin V and PI staining and visualization by flow cytometry and compared to an untreated population shown in E.

Previous studies have shown that the addition of IL-4 to CLL culture enhances in vitro survival by inducing resistance to apoptosis [12, 13]. In this study we confirmed that the addition of IL-4 to CLL cell cultures resulted in a significant reduction in spontaneous apoptosis after 48h (31.4% to 14.1%, P<0.0001). Co-culture with either NTL or CD40L cells proved even more cytoprotective for CLL cells (Figure 1A; P = 0.005 and P = 0.034 respectively); addition of IL-4 to these co-cultures did not further enhance the cytoprotection (NTL: P = 0.31, CD40L: P = 0.07). As a result of these findings, all subsequent experiments were carried out in suspension cultures supplemented with IL-4 and in co-culture with NTL and CD40L cells unless stated otherwise.

### NVP-AUY922-AG is more potent than fludarabine and retains toxicity in the presence of IL-4 and in NTL and CD40L expressing co-culture

The Hsp90 inhibitor, NVP-AUY922-AG was almost a log more potent than fludarabine in CLL cells cultured in suspension without IL-4. The mean LD_50_ for the Hsp90 inhibitor NVP-AUY922-AG was 0.18μM ± 0.20 and was 1.16 ± 1.74 for fludarabine. Furthermore, NVP-AUY922-AG retained its potency against primary CLL cells even in the presence of IL-4 (Figure 1B, P = 0.43, Table 1.) whereas the cytotoxic effects of fludarabine were significantly attenuated under these conditions (P = 0.008, Table 1). Furthermore, co-culturing CLL cells with NTL or CD40L cells completely abrogated the ability of fludarabine to induce cell death. In contrast, NVP-AUY922-AG retained the ability to kill cells under these cytoprotective conditions (Figure 1C, Table 1, NTL P = 0.85, CD40L P = 0.11). Figure 1D shows a composite dose-response curve for 3 individual CLL patients. We went on to confirm that NVP-AUY922-AG-induced cytotoxicity was mediated via apoptosis and this was apparent even at concentrations as low as 0.01μM (Figure 1E).

**Table 1:** 

	IL-4	n	NVP-AUY922-AG LD_50_ μM ± SD	n	Fludarabine LD_50_ μM ± SD
**Suspension**	−	9	0.18 ± 0.20	13	1.16 ± 1.74
	+	26	1.25 ± 3.54	25	13.12 ± 20.89
**NTL**	−	3	0.14 ± 0.06	4	>100 ± -
	+	22	1.41 ± 1.60	32	>100 ± -
**CD40L**	−	3	0.27 ± 0.15	4	>100 ± -
	+	24	5.791 ± 13.75	32	>100 ± -

### NVP-AUY922-AG depletes AKT, IKKα and IKKβ proteins

**Figure 2:**
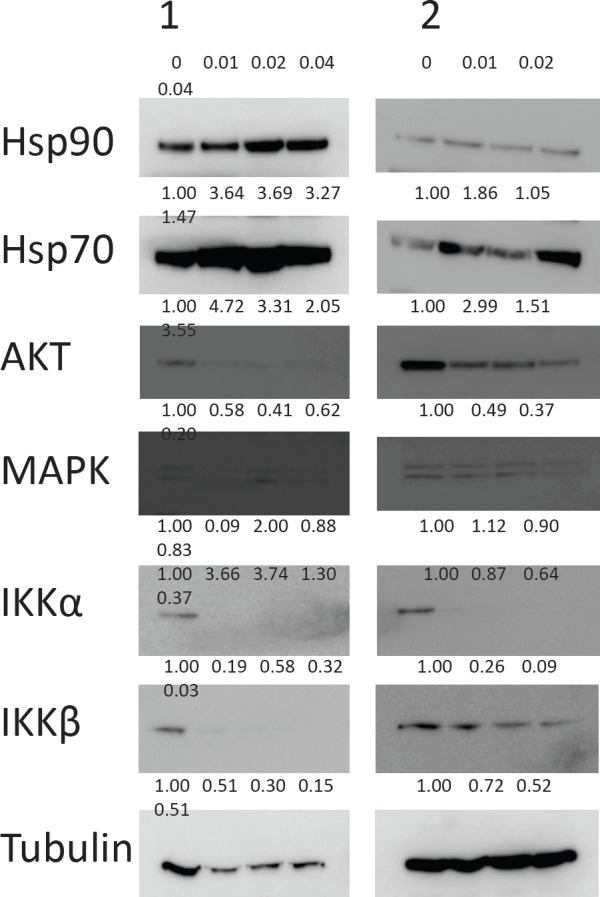
Known Hsp90 client proteins AKT, IKKα and IKKβ are depleted by NVP-AUY922-AG Two representative samples are shown of CLL cells treated with NVP-AUY922-AG in vitro. Western blotting showed depletion of AKT, IKKα and IKKβ proteins in a concentration dependent manner (μM) after treatment with NVP-AUY922-AG for 24h. Images were quantified by densitometry and the level of expression relative to untreated control and tubulin expression if shown under each band of protein. Expression of the MAPK protein was not appreciably decreased by NVP-AUY922-AG while expression of both Hsp90 and Hsp70 proteins was increased.

We went on to investigate the effects of NVP-AUY922-AG on number of known pro-survival Hsp90 client proteins AKT, IKKα and IKKβ [8, 14, 15]. All three proteins were depleted in CLL cells treated with NVP-AUY922-AG for 24h in a concentration-dependent manner (Figure 2). In contrast, MAPK, another Hsp90 client protein, was not substantially decreased at low NVP-AUY922-AG concentrations (0.01-0.04μM) suggesting a degree of selectivity for NVP-AUY922-AG (Figure 2). In keeping with previous studies, [16, 17] the inhibition of Hsp90 resulted in an increase in Hsp70 protein expression (Figure 2). It is worthy of note that Hsp90 itself was not diminished in our assay system but NVP-AUY922-AG inhibits ATPase activity at the N-terminus of Hsp90 thereby promoting client protein dissociation rather than Hsp90 protein degradation [18].

### NVP-AUY922-AG is highly synergistic with fludarabine

**Figure 3:**
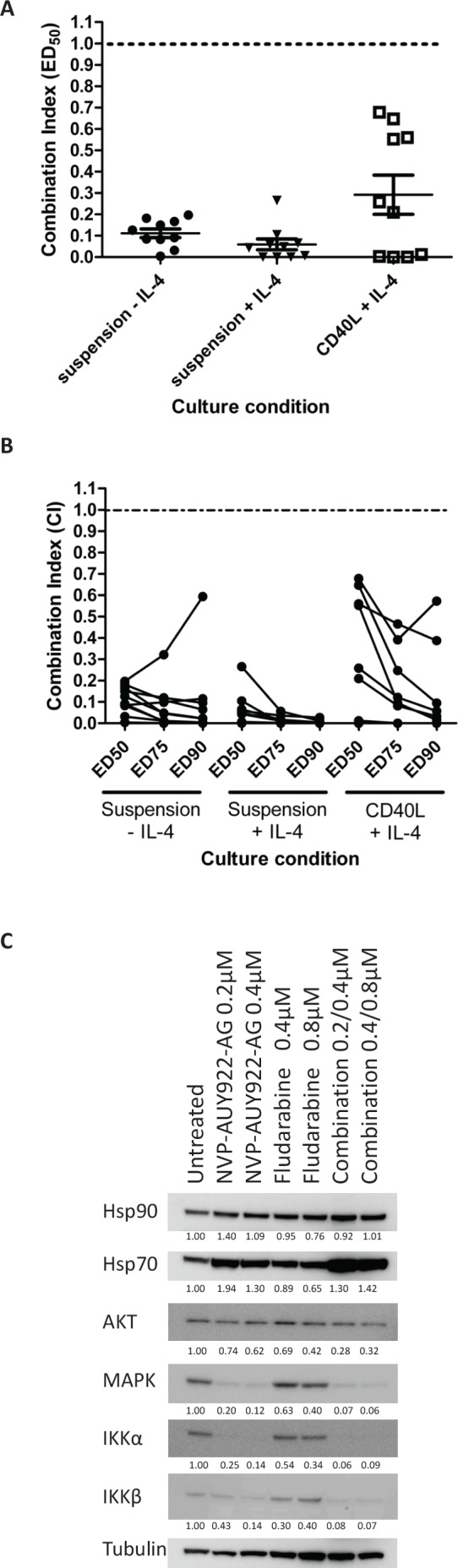
NVP-AUY922-AG is strongly synergistic with fludarabine and Akt, IKKα and IKKβ are strongly depleted by NVP-AUY922-AG and fludarabine in combination A) NVP-AUY922-AG and fludarabine were combined at a constant ratio (1:2) in vitro and the CI calculated at the ED_50_ concentration calculated were CI < 1 indicates synergy between NVP-AUY922-AG and fludarabine. Strongly synergy between the two drugs was indicated in suspension culture and in the cytoprotective CD40L expressing culture conditions. Samples refractory to fludarabine in vitro showed synergy between the two drugs in all culture conditions. B) Synergy was shown over a range of concentrations of the drugs used. In the majority of cases, the level of synergy appeared to be stronger towards higher ED values. C) NVP-AUY922-AG and fludarabine result in the depletion of expression of AKT, MAPK, IKKα and IKKβ proteins. The effect of fludarabine as a single agent on expression of all four proteins is smaller than that induced by NVP-AUY922-AG. Combination of Fludarabine and NVP-AUY922-AG produced a larger decrease in expression of these proteins enforcing the mechanism of synergy between the drugs at a molecular level. Quantitation of the level of protein expression is shown below the relevant blot and is relative to α-tubulin expression.

Given that fludarabine-based regimens are the standard of care for the treatment of CLL [19-21], we assessed the effects of combining fludarabine with NVP-AUY922-AG in vitro. The combination of these drugs resulted in remarkably strong synergy in the absence of IL-4 and this was further enhanced by the addition of IL-4 to the cultures (Figure 3A, mean CI at LD_50_; -IL-4 = 0.11±0.06, +IL-4 = 0.06±0.08). Furthermore, the synergistic interaction between NVP-AUY922-AG and fludarabine was observed over a wide range of concentrations in all the culture conditions tested (Figure 3B). Given that we have shown that fludarabine resistance is markedly increased in the presence of IL-4, it seems likely that the increased synergy seen under these conditions is promoted, at least in part, by the ability of NVP-AUY922-AG to reverse IL-4-mediated fludarabine resistance mechanisms.

In an attempt to investigate the mechanistic basis of the synergy observed between NVP-AUY922-AG and fludarabine, we examined the effect of both agents, alone and in combination, on the expression of AKT, MAPK, IKKα and IKKβ proteins in CLL cells. Both NVP-AUY922-AG and fludarabine reduced the expression of all four proteins at concentrations employed in the synergy experiments (Figure 3C). The decrease in these proteins was enhanced when the agents were used in combination. In contrast, Hsp90 protein expression was not altered by the combination of NVP-AUY922-AG and fludarabine. Hsp70 protein expression was increased by NVP-AUY922-AG and NVP-AUY922-AG combined with fludarabine but was slightly decreased by fludarabine alone.

### NVP-AUY922-AG inhibits NF-κB target gene transcription

**Figure 4:**
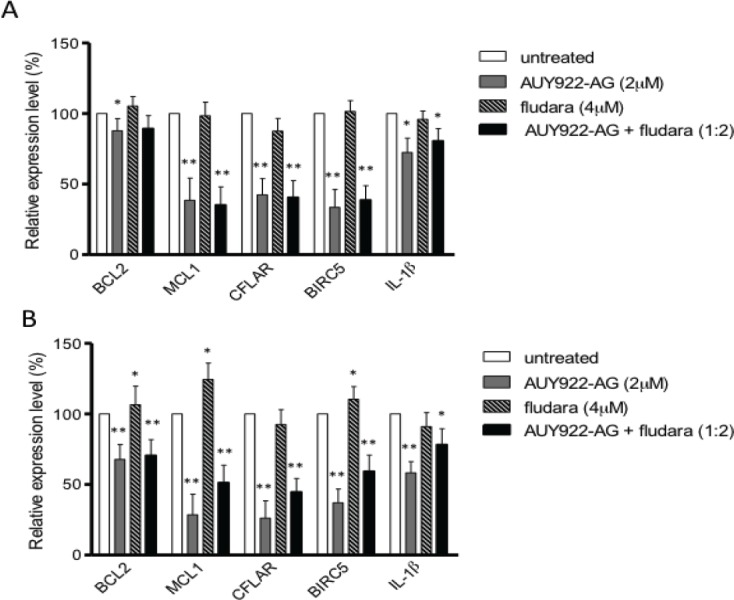
NF-κB regulated genes are inhibited by NVP-AUY922-AG Anti-apoptotic genes BCL2, MCL1, CFLAR, BIRC5 and inflammatory cytokine IL-1β are all regulated by NF-κB. Real-time reverse-transcription PCR was used to measure the relative level of target gene mRNA expression compared to housekeeping gene RPS14 following treatment with NVP-AUY922-AG, fludarabine and both in combination. A) 4h of treatment with NVP-AUY922-AG (2μM) and NVP-AUY922-AG with fludarabine significantly inhibited MCL1, CFLAR and BIRC5. Fludarabine alone did not reduce the mRNA level of these genes. B) MCL1, CFLAR, BIRC5 and IL-1β mRNA levels were all reduced by 24h treatment with NVP-AUY922-AG or NVP-AUY922-AG and fludarabine in combination. Fludarabine as a single agent induced mRNA expression of MCL1 and BIRC5. * P < 0.05, ** P < 0.0001.

Given the inhibitory effects of NVP-AUY922-AG on IKKα and IKKβ, we next investigated genes that are transcriptionally regulated by NF-κB. In particular we investigated transcriptional changes in the anti-apoptotic genes BCL2, MCL1, CFLAR and BIRC5 as well as the pro-inflammatory cytokine IL-1β. We measured the relative level of target gene mRNA expression, when compared with the house-keeping gene RPS14, following treatment of primary CLL cells with NVP-AUY922-AG, fludarabine and the combination for 4 hours and 24 hours. Transcription of the short half-life genes MCL1, CFLAR and BIRC5 were significantly inhibited at 4 hours following treatment with 2μM NVP-AUY922-AG and the combination of NVP-AUY922-AG and fludarabine (Figure 4A). In contrast, treatment with 4μM fludarabine as a single agent did not significantly reduce the transcription of these genes at the 4-hour time point. A similar pattern of expression was observed following exposure to drug for 24 hours; all of the NF-κB regulated genes were significantly inhibited following exposure to NVP-AUY922-AG and the combination of NVP-AUY922-AG and fludarabine (Figure 4B). Importantly, exposure to fludarabine alone appeared to induce the transcription of MCL1 and BIRC5 at 24 hours consistent with the view that these genes may play a role in facilitating fludarabine resistance in vitro [1, 22]. Furthermore, the combination of NVP-AUY922-AG and fludarabine suppressed the fludarabine-mediated induction of MCL1 and BIRC5. It is therefore conceivable that that NVP-AUY922-AG-mediated inhibition of anti-apoptotic genes would increase the sensitivity of primary CLL cells to the effects of fludarabine in the combination thereby contributing to the synergy seen with these agents.

## DISCUSSION

CLL is an incurable disease using the standard therapeutic options currently available [23, 24]. Although most patients initially respond to chemotherapy, they invariably relapse and develop drug resistance. There is growing evidence that resistance arises in the pro-survival microenvironments found in the lymph nodes and bone marrow [25] and both cellular and humoral interactions in these tissue sites likely play a role in this process [26]. Recent evidence suggests that the lymph node interactions preferentially induce CLL cell activation and proliferation when compared to the bone marrow microenvironment [2] and this is associated with the activation of the NF-κB pathway. NF-κB target genes are involved in cell-cycle regulation, inhibition of apoptosis, signal transduction and chemotaxis [2]. In the lymph node microenvironment CD40-CD40L interactions between CLL cells and activated T-cells induces NF-κB, drives CLL cell proliferation and promotes resistance to chemotherapeutics [27]. Consequently, recent research has focused on targeting the microenvironment and the signaling pathways within CLL cells that it activates [25]. However, cancer cells are adept at circumventing pharmacological blockade of individual signaling pathways so the concept of multiple targeted approaches appears rational (24). In this context, inhibition of Hsp90 is an attractive possibility in CLL and other cancers [28-31] as Hsp90 is a molecular chaperone that acts to ensure the correct folding of nascent proteins and their maintenance or targeting for degradation by the proteasome. Inhibition of Hsp90 has been reported to simultaneously down regulate a number of client proteins that are involved in signaling pathways implicated in cancer [28, 31].

Given the importance of microenvironmental effects on CLL cells, it is increasingly clear that potential treatments for CLL should be evaluated under conditions that mimic these pro-survival niches. Therefore, in this study we added IL-4 to liquid culture conditions and used mouse fibroblast co-cultures which promote the up-regulation of co-stimulatory and adhesion molecules in the CLL cells [32]. Both of these approaches significantly reduced the level of spontaneous apoptosis seen after 48h [33, 34]. The addition of IL-4 to liquid culture conditions induced marked resistance to fludarabine. Resistance to apoptosis induced by fludarabine has previously been described in CLL cells cultured with CD40L-expressing cells in the presence of IL-4.[35] The mechanism(s) for this have not been fully elucidated but the upregulation of Bcl-2 may well be a contributory factor.[33] In contrast, NVP-AUY922-AG was equipotent in IL-4 supplemented cultures suggesting that its mechanism of killing was distinct from fludarabine. Furthermore, under co-culture conditions on NTL of CD40L cells fludarabine was even less cytotoxic whereas NVP-AUY922-AG retained its cell killing activity. The cell killing activity of NVP-AUY922-AG in the co-culture systems is promising as it suggests that this agent can overcome the survival signals induced in the CLL cells by interactions with the microenvironment including CD40-CD40L signaling and protection by IL-4 [36].

At the molecular level, we demonstrated Hsp90 inhibition resulted in an increase in Hsp70; a finding consistent with previously published reports of Hsp90 inhibitors.[16, 17] It is worthy of note that the induction of Hsp70 expression as a result of Hsp90 inhibition may be problematic as Hsp70 is a cytoprotective protein that can block both caspase-dependent and caspase-independent apoptosis, and also autophagic cell death and necrosis [28]. However, the stability and function of multiple client proteins are regulated by Hsp90 and these proteins function as regulators of cell growth, differentiation and in apoptotic pathways[37]. Consequently, inhibition of Hsp90 function by NVP-AUY922-AG resulted in decreased expression of AKT, MAPK, IKKα and IKKβ. This confirms that inhibition of Hsp90 targets multiple signaling pathways. [14, 15] Of particular interest is the inhibition of IKK and IKKβ as these modulate expression of NF-κB through phosphorylation of IκB leading to IκB degradation and subsequent release and activation of NF-κB.[38, 39] NF-κB has been previously shown to be a prognostic marker and a therapeutic target in CLL [9, 11, 40] and the ability to suppress NF-κB activation in CLL cells may be critical to the success of a treatment. We have recently shown that NF-κB activation is higher in previously treated CLL patients [40], which in turn makes the cells impervious the effects of other therapies [41].

The most striking finding of this study was the remarkable cytotoxic synergy seen in CLL cells treated with NVP-AUY922-AG and fludarabine, particularly in cytoprotective culture conditions (Figure 3). This synergy was mirrored at the molecular level as the inhibition of AKT, IKKα and IKKβ by NVP-AUY922-AG alone was enhanced when NVP-AUY922-AG was used in combination with fludarabine. Additionally, co-culture of CLL cells with CD40L-expressing cells results in the up-regulation of anti-apoptotic genes including NF-κB target genes BCL2 and MCL1 [42, 43], CFLAR and BIRC5 [44]. Quantitative PCR analysis showed that NVP-AUY922-AG resulted in inhibition of expression of MCL1, CFLAR and BIRC5 in primary CLL cells (Figure 4). In contrast, fludarabine induced the transcription of MCL1 and BIRC5. Importantly, the combination of NVP-AUY922-AG and fludarabine resulted in the net transcriptional repression of these genes perhaps providing a molecular explanation for the synergy observed.

In conclusion, inhibition of Hsp90 appears to be an attractive prospective for therapy in CLL as it targets multiple intracellular signaling pathways that are preferentially activated in cytoprotective microenvironments in vivo. Encouragingly, NVP-AUY922-AG retained its activity in the presence of such signals in vitro and showed remarkable synergy with fludarabine under the same conditions. As such, early phase trials of this agent, both alone and in combination, would seem warranted.

## MATERIALS AND METHODS

### Cell isolation and culture

Lymphocytes from peripheral blood samples from CLL patients were separated using Ficoll-Hypaque (Sigma, Poole, UK). Samples were washed in phosphate buffered saline (PBS) and counted. Patients were diagnosed using a combination of clinical criteria and immunophenotyping. Informed consent was obtained in accordance with the ethical approval obtained from South East Wales Research Ethics Committee (02/4806) prior to samples being taken. Separated lymphocytes were maintained in RPMI medium supplemented with 10% fetal bovine serum (FBS), penicillin (50U/ml) and streptomycin (50μg/ml). Where indicated, recombinant human IL-4 (R and D Systems, Abingdon, UK) (5ng/ml) was added to the culture medium (5ng/ml). Mouse embryonic fibroblast L-cells, either non-transfected (NTL) or L-cells expressing CD40 ligand (CD40L) [45] were used where indicated as feeder layers.

### Toxicity assays

The toxicity of the Hsp90 inhibitor NVP-AUY922-AG in primary CLL cells was determined in cells in suspension culture, on NTL and CD40L feeder layers over 48h both in the presence and absence of IL-4. NVP-AUY922-AG was tested at concentrations between 0.008μM and 50μM. Sensitivity to fludarabine (0.15μM to 10μM) was also measured in the same samples. CLL cells co-cultured on a feeder layer were pretreated with NVP-AUY922-AG or fludarabine for 2 hours before being placed on the feeder layer to preserve the integrity of the layer and ensure that only the CLL cells were exposed to drug. Cell viability was assessed by flow cytometry.

### Synergy

Synergy between NVP-AUY922-AG and fludarabine was tested in suspension culture (± IL-4) and on CD40L cells (with IL-4) over 48h. A constant molar ratio of NVP-AUY922-AG 1:2 fludarabine was used based on the relative activities of both agents determined in the preceding toxicity experiments. Concentrations used were NVP-AUY922-AG (0.001 to 50μM) and fludarabine (0.002 to 100μM). Cell death was assessed on an Accuri C6 flow cytometer (BD Accuri Cytometers, Ann Arbor, MI, USA). Synergy was calculated according to the median effect method using CalcuSyn software [46].

### Annexin V positivity

CLL lymphocytes treated with NVP-AUY922-AG (0.01, 0.02, 0.04, 2.0 and 4.0μM) for 48h were tested for annexin V positivity using an Annexin V apoptosis detection kit (Axxora Ltd, Matford Court, UK) according to the manufacturer's instructions. Untreated cells were used a control. Cells treated with NVP-AUY922-AG were maintained in suspension culture with IL-4.

### Immunoblotting

Equal numbers of cells (5 × 10^6^ cells) treated with NVP-AUY922-AG (0, 0.01, 0.02 and 0.04μM) in suspension culture with IL-4 for 24h were washed with PBS and lyzed by resuspension in lysis buffer (HEPES 50mM, sodium fluoride 5mM, iodoacetamide 5mM, sodium chloride 75mM, NP40 1%, PMSF 1mM, sodium orthovanadate 1mM, protease inhibitors (Sigma) 1%, phospatase inhibitor cocktail 2 (Sigma) 1%, phosphatase inhibitor cocktail 3 (Sigma) 1%) for 30 minutes at 4°C followed by centrifugation at 16 000 × g. Clarified lyzates were subjected to electrophoresis using NuPage precast 4-12% Bis-Tris gels (Invitrogen, Paisley, UK) followed by transfer to PVDF membranes (GE Healthcare UK Ltd, Little Chalfont, UK). Immunoblotting was performed with antibodies to Hsp90, Akt, mitogen-activated protein kinase (MAPK), IκB kinase α (IKKα), IκB kinase β (IKKβ) (New England Biolabs (UK) Ltd, Hitchin, UK), Hsp70 (Millipore (UK) Ltd, Watford, UK), Tubulin (Abcam, Cambridge, UK). Cells treated with NVP-AUY922-AG and fludarabine in combination (NVP-AUY922-AG 0.02 and 0.04μM, fludarabine 2.0 and 4.0 μM) in suspension culture with IL-4 for 24h at the ratio previously described were also lyzed and immunoblotted with the same panel of antibodies. Additionally, cells treated with NVP-AUY922-AG on CD40L cells with IL-4 for 24h were also lyzed and immunoblotted for the same panel of antibodies.

### Real-time reverse transcription-PCR

Untreated cells and cells treated with NVP-AUY922-AG and fludarabine in combination (NVP-AUY922-AG 2.0μM, fludarabine 4.0μM and both drugs at a ratio of 1:2) for 4 and 24h. 5x10^6^ CLL cells were re-suspended in 1ml Trizol reagent and RNA was extracted using chloroform and isopropanol. RNA (1 μg) was used in a 20μL reverse transcription (RT) reaction containing 10× Buffer II, 5 mmol/L MgCl_2_, 0.5 μmol/L deoxynucleotide triphosphates, 2.5 units reverse transcriptase, 1 unit RNase inhibitor, and 2.5 μmol/L random hexamers. cDNA (2 μL) was placed into the RT-PCR reaction. SYBR Green technology (Roche Diagnostics, Burgess Hill, UK) was used to quantify the amount of RNA present in each sample using primer pairs for BCL2, MCL1, CFLAR, BIRC5, IL-1β and RPS14. All primers were purchased from Eurogentec Ltd (Southampton, UK). The amount of mRNA was assessed using real-time RT-PCR using the LightCycler System (Roche Diagnostics). The amount of RPS14 mRNA was quantified in all samples as an internal house-keeping control, and the results of the real-time RT-PCR were expressed as normalized target gene values (e.g. the ratio between BCL2 and RPS14 transcripts calculated from the crossing points of each gene). All experiments were performed in duplicate. Total RNA was amplified using the following primers:

BCL2: 5′-aagattgatgggatcgttgc-3′ (forward) and 5′-tgtgctttgcattcttggac-3′ (reverse);
MCL1: 5′-aaaagcaagtggcaagagga-3′ (forward) and 5′-ttaatgaattcggcgggtaa-3′ (reverse);
CFLAR: 5′-gtggagacccacctgctca-3′ (forward) and 5′-ggacacatcagatttatccaaatcc-3′ (reverse);
BIRC5: 5′-ttagcagaaaatgcactccag-3′ (forward) and 5′-ctggttttaaggatggccttt-3′ (reverse);
IL-1β: 5′-tggcagaaagggaacagaaa-3′ (forward) and 5′-acttcttgccccctttgaat-3′ (reverse);
RPS14: 5′-ggcagaccgagatgaactct-3′ (forward) and 5′-ccaggtccaggggtcttggt-3′ (reverse).
